# Validation of the Antioxidant and Enzyme Inhibitory Potential of Selected Triterpenes Using In Vitro and In Silico Studies, and the Evaluation of Their ADMET Properties

**DOI:** 10.3390/molecules26216331

**Published:** 2021-10-20

**Authors:** Nilufar Z. Mamadalieva, Fadia S. Youssef, Hidayat Hussain, Gokhan Zengin, Adriano Mollica, Nawal M. Al Musayeib, Mohamed L. Ashour, Bernhard Westermann, Ludger A. Wessjohann

**Affiliations:** 1 Institute of the Chemistry of Plant Substances, Academy Sciences of Uzbekistan, Tashkent 100170, Uzbekistan; 2 Department of Bioorganic Chemistry, Leibniz Institute of Plant Biochemistry, Weinberg 3, 06120 Halle (Saale), Germany; hidayat.hussain@ipb-halle.de (H.H.); bernhard.westermann@ipb-halle.de (B.W.); ludger.wessjohann@ipb-halle.de (L.A.W.); 3 Department of Pharmacognosy, Faculty of Pharmacy, Ain Shams University, Cairo 11566, Egypt; fadiayoussef@pharma.asu.edu.eg (F.S.Y.); ashour@pharma.asu.edu.eg (M.L.A.); 4 Department of Biology, Science Faculty, Selcuk University, Konya 42130, Turkey; gokhanzengin@selcuk.edu.tr; 5 Department of Pharmacy, University “G. d’Annunzio” of Chieti-Pescara, 66100 Chieti, Italy; a.mollica@unich.it; 6 Department of Pharmacognosy, College of Pharmacy, King Saud University, Riyadh 11495, Saudi Arabia; nalmusayeib@ksu.edu.sa

**Keywords:** antioxidants, enzyme inhibition, in vitro assays, triterpenes, virtual screening, inflammation, Alzheimer’s disease

## Abstract

The antioxidant and enzyme inhibitory potential of fifteen cycloartane-type triterpenes’ potentials were investigated using different assays. In the phosphomolybdenum method, cycloalpioside D (**6**) (4.05 mmol TEs/g) showed the highest activity. In 1,1-diphenyl-2-picrylhydrazyl (DPPH*) radical and 2,2′-azino-bis(3-ethylbenzothiazoline)-6-sulfonic acid (ABTS) cation radical scavenging assays, cycloorbicoside A-7-monoacetate (**2**) (5.03 mg TE/g) and cycloorbicoside B (**10**) (10.60 mg TE/g) displayed the highest activities, respectively. Oleanolic acid (**14**) (51.45 mg TE/g) and 3-*O*-β-d-xylopyranoside-(23*R*,24*S*)-16β,23;16α,24-diepoxycycloart-25(26)-en-3β,7β-diol 7-monoacetate (**4**) (13.25 mg TE/g) revealed the highest reducing power in cupric ion-reducing activity (CUPRAC) and ferric-reducing antioxidant power (FRAP) assays, respectively. In metal-chelating activity on ferrous ions, compound **2** displayed the highest activity estimated by 41.00 mg EDTAE/g (EDTA equivalents/g). The tested triterpenes showed promising AChE and BChE inhibitory potential with 3-*O*-β-d-xylopyranoside-(23*R*,24*S*)-16β,23;16α,24-diepoxycycloart-25(26)-en-3β,7β-diol 2′,3′,4′,7-tetraacetate (**3**), exhibiting the highest inhibitory activity as estimated from 5.64 and 5.19 mg GALAE/g (galantamine equivalent/g), respectively. Compound **2** displayed the most potent tyrosinase inhibitory activity (113.24 mg KAE/g (mg kojic acid equivalent/g)). Regarding α-amylase and α-glucosidase inhibition, 3-*O*-β-d-xylopyranoside-(23*R*,24*S*)-16β,23;16α,24-diepoxycycloart-25(26)-en-3β,7β-diol (**5**) (0.55 mmol ACAE/g) and compound **3** (25.18 mmol ACAE/g) exerted the highest activities, respectively. In silico studies focused on compounds **2**, **6**, and **7** as inhibitors of tyrosinase revealed that compound **2** displayed a good ranking score (−7.069 kcal/mole) and also that the ΔG free-binding energy was the highest among the three selected compounds. From the ADMET/TOPKAT prediction, it can be concluded that compounds **4** and **5** displayed the best pharmacokinetic and pharmacodynamic behavior, with considerable activity in most of the examined assays.

## 1. Introduction

Free radicals are highly active components that are generated naturally within the human body. They may cause many adverse effects manifested by the oxidation of lipids, proteins, and DNA. These harmful effects are limited by the presence of the antioxidant system that effectively protects the living organism. This protective system includes antioxidant enzymes represented by peroxidase, catalase, superoxide dismutase, glutathione, and thioredoxin. In addition, non-enzymatic antioxidants are comprised of retinol (vitamin A), ascorbic acid (vitamin C), tocopherol (vitamin E), uric acid, and glutathione. The disturbance in the balance between the antioxidant defense and the generation of free radicals causes oxidative stress [1].

This oxidative stress that mainly results from the overproduction of reactive oxygen species (ROS) triggers many hazardous disorders including cancer, cardiovascular diseases, atherosclerosis, obesity, diabetes, and inflammatory diseases [2,3]. Diabetes mellitus is a serious metabolic disease characterized by hyperglycemia, polydipsia, polyphagia, and frequent urination that attacks nearly 10% of the population worldwide [4]. Moreover, Alzheimer′s disease (AD) is an irreversible neurological disorder that occurs progressively and its incidence is consequently elevated with age [5].

Meanwhile, naturally occurring antioxidants counteract oxidative stress to a great extent, showing a wide range of biological activities. They effectively prohibited oxidative stress-associated disorders including neurodegenerative disorders and diabetes as well [6,7,8,9]. Hence, phytoconstituents derived from different organisms, but mostly from plants, have become the focus of many types of antioxidant and anti-inflammatory research studies for nutraceutical and drug discovery, e.g., as lead entities adopted by pharmaceutical companies. The plant kingdom constitutes a hub for highly popular natural antioxidants, represented mainly by ascorbate and polyphenolic compounds such as flavonoids, tocopherols, and phenolic terpenoids. Furthermore, these phytochemicals afford many other activities that can relieve many diseases in addition to their pronounced antioxidant potential [10,11,12].

Terpenes represent a large category of naturally occurring compounds possessing significant biological activities. Many polycyclic triterpenes, especially sterols, are biosynthesized via squalene epoxide arrangements in a chair-chair-chair-boat manner that is consequently followed by condensation. Terpenes include several classes represented by mono and sesquiterpene components that prevail in essential oils, diterpenes, and triterpenes (with steroids) of different types, as well as in tetraterpene carotenoids and polyterpenes. Nowadays, there is an increasing interest in natural triterpenoids due to their outstanding biological activities as exemplified by reported anticancer, antiviral, bactericidal, spermicidal, anti-allergic, fungicidal, and cardiovascular protective effects [13,14,15,16,17].

Triterpenes are classified into many subclasses per their chemical structure. The main groups of triterpenoids and their glycosides are represented by tetracyclic derivatives of the cycloartane, euphane, protostane, and dammarane type, in addition to the pentacyclic derivatives of the gammacerane, hopane, ursane, and lupine type. This study aimed to investigate the antioxidant and enzyme inhibitory potential of fifteen triterpenes. The antioxidant activity was determined using different assays, including total antioxidant activity by the phosphomolybdenum method; 1,1-diphenyl-2-picrylhydrazyl (DPPH*) radical scavenging activity, 2,2′-azinobis(3-ethylbenzothiazoline)-6-sulfonic acid (ABTS) cation radical scavenging activity, cupric ion-reducing activity (CUPRAC), and the ferric-reducing antioxidant power (FRAP) assay; as well as metal-chelating activity on the ferrous ions assay. Furthermore, the enzyme inhibitory potential was investigated via determining the inhibitory potential of the fifteen triterpenes on acetylcholinesterase, butyrylcholinesterase, tyrosinase, α-amylase, and α-glucosidase enzymes. Additionally, the examined triterpenes were subjected to molecular modelling studies on tyrosinase enzymes, followed by MM-GBSA (molecular mechanics energies combined with generalized Born and surface area continuum solvation) calculations of the obtained docking poses. Furthermore, the prediction of ADMET (absorption, distribution, metabolism, excretion, and toxicity) properties as well as TOPKAT (toxicity prediction) for all of the examined molecules was performed using Discovery Studio 4.5 software (Accelrys Inc., San Diego, CA, USA).

## 2. Results and Discussion

### 2.1. Triterpenes Used in This Study

**Figure 1:**
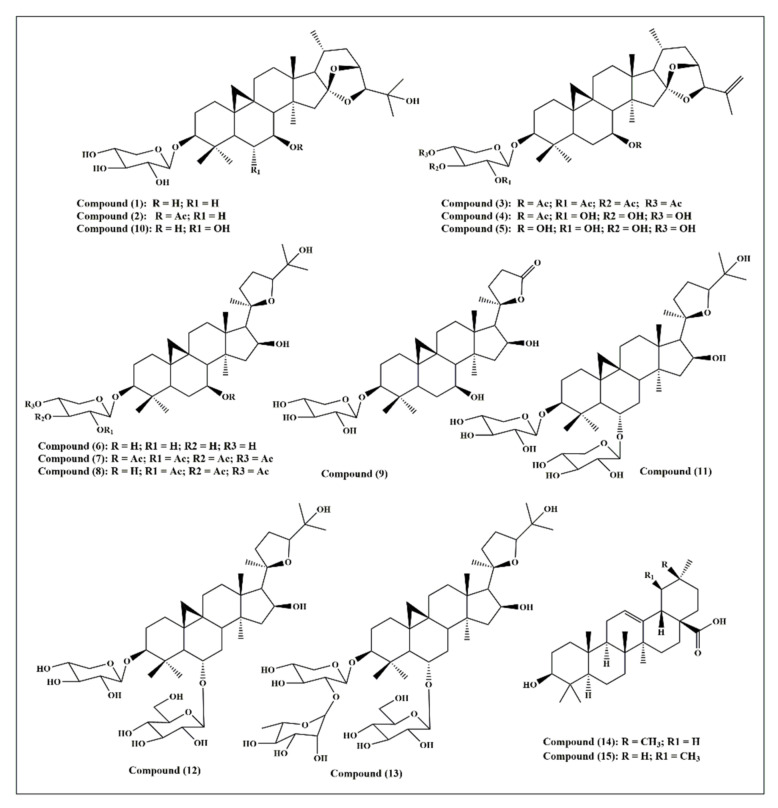
A scheme showing the fifteen triterpene compounds selected for this study.

Fifteen triterpenes were used in this study and they are illustrated in Figure 1: cycloorbicoside A (**1**), cycloorbicoside A-7-monoacetate (**2**), 3-*O*-β-d-xylopyranoside-(23*R*,24*S*)-16β,23;16α,24-di-epoxycycloart-25(26)-en-3β,7β-diol2′,3′,4′,7-tetraacetate (**3**), 3-*O*-β-d-xylopyranoside-(23*R*,24*S*)-16β,23; 16α,24-diepoxycycloart-25(26)-en-3β,7β-diol 7–monoacetate (**4**), 3-*O*-β-d-xylopyranoside-(23*R*,24*S*)-16β,23;16α,24-diepoxycycloart-25(26)-en-3β,7β-diol (**5**), cycloalpioside D (**6**), cycloalpioside D-2′,3′,4′,7-tetraacetate (**7**), cycloalpioside D-2′,3′,4′-triacetate (**8**), 3-*O*-β-d-xylopyronoside-20*R*-25-norcycloartan-3β,7β,16β-triol-20,24-olide (**9**), cycloorbicoside B (**10**), cyclosiversioside E (**11**), astragaloside IV (cyclosieversioside F or astrasieversianin XIV) (**12**), cyclosiversioside H (**13**), oleanolic acid (**14**), and ursolic acid (**15**).

### 2.2. In Vitro Assays for the Evaluation of the Antioxidant Activity of the Studied Triterpenes

#### 2.2.1. Total Antioxidant Activity by the Phosphomolybdenum Method

The phosphomolybdenum method was used to evaluate the total antioxidant activity of all the compounds studied. The molybdenum ion-reducing potential of various compounds is illustrated in Table 1. Basically, Mo(VI) is reduced to Mo(V) by the action of antioxidant compounds that results in the formation of the green phosphate/Mo(V) complex as a byproduct, which is detected spectrophotometrically at λ = 695 nm. Compound **6** (4.05 mmol TEs/g), followed by compounds **2** (2.26 mmol TEs/g), **3** (1.98 mmol TEs/g), and **11** (1.90 mmol TEs/g), showed the highest total antioxidant activity. In contrast, compound **14** revealed the lowest activity in this assay (0.2 mmol TEs/g; Table 1).

#### 2.2.2. 1,1-Diphenyl-2-Picrylhydrazyl (DPPH*) Radical Scavenging Capacity Assay

**Figure 2:**
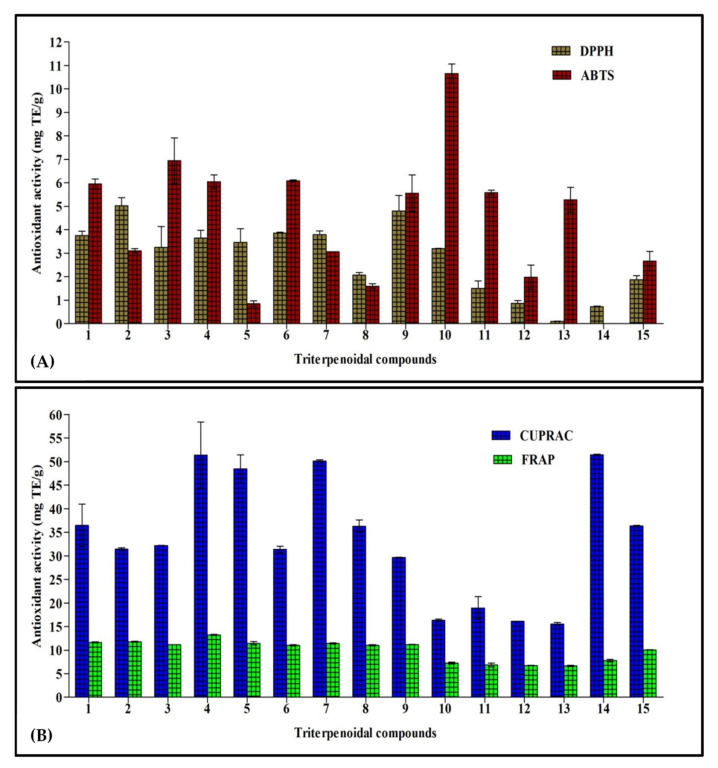
Antioxidant activity of fifteen triterpenes, employing DPPH and ABTS (**A**), and CUPRAC and FRAP (**B**) expressed in mg TE/g. Values are reported as mean ± S.D.; TE: Trolox equivalents.

This assay depends on the bleaching of the purple color of the DPPH methanol solution due to the electron or hydrogen atom donating capability of the tested sample that causes a reduction in the radical solutions and is directly correlated to the antioxidant ability of the tested sample [18]. Results illustrated in Figure 2A show that cycloorbicoside A-7-monoacetate (**2**) (5.03 mg TE/g) showed the highest activity, followed by compounds **9** (4.80 mg TE/g), **6** (3.86 mg TE/g), and **7** (3.79 mg TE/g). In contrast, cyclosiversioside H (**13**) revealed the lowest activity (0.10 mg TE/g; Figure 2A).

#### 2.2.3. 2,2′-Azinobis(3-Ethylbenzothiazoline)-6-Sulfonic Acid (ABTS) Cation Radical Scavenging Activity

In this assay, the colored free radical 2,2′-azino-bis(3-ethylbenzthiazoline-6-sulfonic acid) radical (ABTS^+^) was greatly employed to evaluate the capability of a compound to transfer electrons, which is widely correlated to the presence of antioxidants in the solution. Cycloorbicoside B (**10**) displayed the highest activity in this assay (10.60 mg TE/g), followed by the compounds **3** (6.95 mg TE/g), **6** (6.08 mg TE/g), and **4** (6.05 mg TE/g). At the other end, oleanolic acid (**14**) revealed no activity in this assay (Figure 2A).

#### 2.2.4. Cupric Ion-Reducing Activity (CUPRAC) Assay

Reducing power is frequently adopted as an indicator for donating electrons, which represents one of the most important mechanisms of antioxidants. The CUPRAC assay determines the reduction of cupric (Cu^2+^)-neocuproine to cuprous ion (Cu^+^). In this assay, oleanolic acid (**14**) revealed the highest reducing power, as evidenced by the results of the CUPRAC assay with activity equaling to 51.45 mg TE/g. This was followed by compounds **4** and **7** that displayed a reducing activity estimated at 51.4 and 50.1 mg TE/g, respectively, while cyclosiversioside H (**13**) revealed the lowest activity (15.5 mg TE/g; Figure 2B).

#### 2.2.5. Ferric-Reducing Antioxidant Power (FRAP) Assay

FRAP is a simple, fast, and reliable method that estimates the reduction of ferric ion (Fe^3+^) to ferrous (Fe^2+^), which is highly correlated with the antioxidant potential of phytochemicals and is detected spectrophotometrically. Compound **4** exerted the most potent FRAP activity, showing 13.3 mg TE/g. Meanwhile, all other compounds, namely **1–9**, revealed activity in the range of 11.0–11.8 mg TE/g, followed by ursolic acid (**15**) that showed activity (10.0 mg TE/g). The compound **13** revealed the lowest activity (6.7 mg TE/g; Figure 2B).

#### 2.2.6. Metal-Chelating Activity on Ferrous Ions

Enzyme activity within the human body greatly depends on the presence of transition elements; meanwhile, they contain some unpaired electrons that combine fast with peroxides producing alkoxyl radicals. Thus, antioxidants that perfectly chelate these ions can inhibit their action, providing a reliable mechanism to combat oxidative stress. Herein, the determination of the formation of ferrous ion–ferrozine complexes was used to investigate the metal-chelating potential of the studied triterpenes. Cycloorbicoside A 7-monoacetate (**2**) displayed the highest metal-chelating activity on ferrous ions at 41.0 mg EDTAE/g. This was followed by ursolic acid (**15**), cycloalpioside D-2′,3′,4′,7-tetraacetate (**7**), and 3-*O*-β-d-xylopyranoside-(23*R*,24*S*)-16β,23;16α,24-diepoxycycloart-25(26)-en-3β,7β-diol 7-monoacetate (**4**), displaying activities of 30.2, 25.4, and 21.9 mg EDTAE/g, respectively. However, oleanolic acid (**14**) showed the lowest activity in this assay, which was equal to 3.05 mg EDTAE/g (Table 1).

Most of the tested compounds showed good antioxidant activities in one or more assays but with different preferences. That undoubtedly reflects their different antioxidant activity modes. Some displayed their antioxidant potential through the scavenging of free radicals, as in DPPH and ABTS assays. Meanwhile, others showed a reduction of antioxidant power, as in the FRAP, CUPRAC, and phosphomolybdenum method assays, or acted by chelating the ions in biorelevant transition elements avoiding electron transfer, such as in the ferrous ion assay.

### 2.3. In Vitro Assays for the Evaluation of the Enzyme Inhibitory Activity of the Studied Triterpenes

#### 2.3.1. Cholinesterase (ChE) Inhibitory Activity

**Figure 3:**
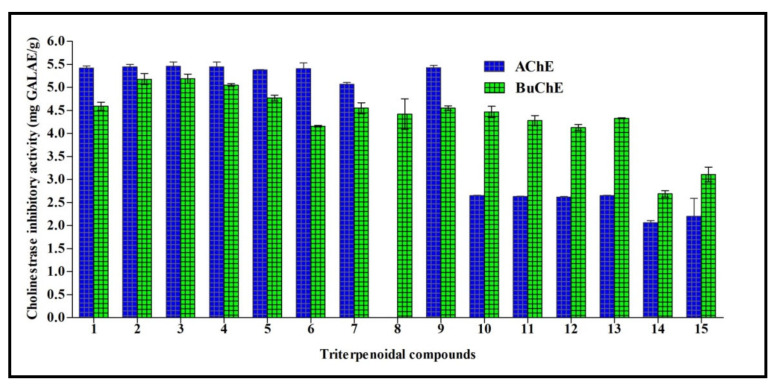
Cholinestrase inhibitory activity of fifteen triterpenes expressed in mg GALAE/g. Values are reported as mean ± S.D.; GALAE: Galatamine equivalent.

Cholinesterasas form a family of enzymes that specifically cleave choline-based esters that mainly act as neurotransmitters. Acetylcholinesterase (AChE) and butyrylcholinesterase (BChE) are the two types of cholinesterase that widely differ according to the cholinergic substrate, with an acetylcholin preference for AChE and butyrylcholine for BChE. Inhibition of cholinesterases results in an elevated level of neurotransmitters and thus can serve as a promising strategy for temporarily alleviating certain neurodegenerative disorders, such as Alzheimer’s disease. All triterpenes tested showed acetylcholinesterase AChE and BChE inhibitory potential, except for compound **8** that had no acetylcholinesterase inhibitory activity. 3-*O*-β-d-Xylopyranoside-(23*R*,24*S*)-16β,23;16α,24-di-epoxycycloart-25(26)-en-3β,7β-diol 2′,3′,4′,7-tetraacetate (**3**) exhibited the highest inhibitory activity for both AChE and BChE, with activities of 5.64 and 5.19 mg GALAE/g, respectively. Regarding AChE, other compounds, namely **1–9** and excluding compound **8**, showed inhibitory activity ranging between 5.07 and 5.45 mg GALAE/g, while compounds **10–15** displayed an inhibitory potential between 2.07 and 2.65 mg GALAE/g. Concerning BChE, most of the compounds showed a considerable inhibitory effect in the range of 4.13–5.18 mg GALAE/g, except compounds **14** and **15** that showed almost equal BChE inhibitory activity (2.06 and 2.20 mg GALAE/g, respectively) (Figure 3).

#### 2.3.2. Tyrosinase Inhibitory Activity

**Figure 4:**
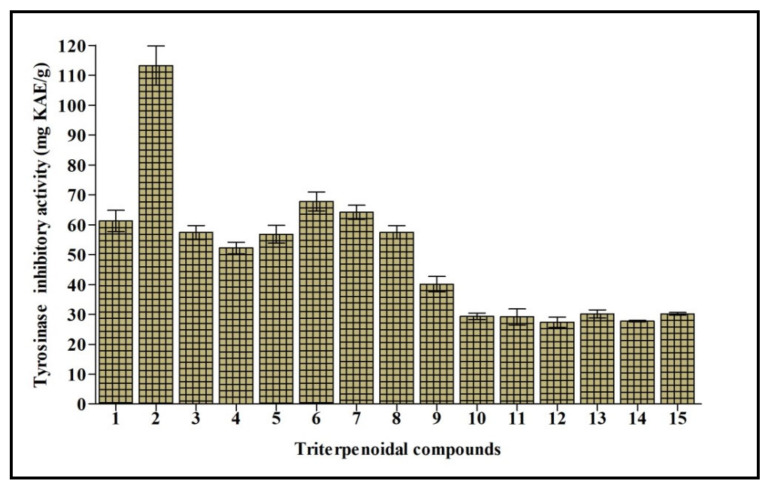
Tyrosinase inhibitory activity of fifteen triterpenes expressed in mg KAE/g. Values are reported as mean ± S.D.; KAE: Kojic acid equivalent. Different letters indicate significant differences in the tested compounds (*p* < 0.05).

Tyrosinase is a polyphenol oxidase enzyme that contains copper and greatly influences the process of melanogenesis. It changes l-tyrosine into l-DOPA with consequent oxidation to dopachrome that stimulates the generation of melanin. Melanin is crucial to counteract the damage caused to hair, skin, and eyes triggered by UV. However, its overproduction is accompanied by the appearance of freckles, melisma, and neurodegenerative disorders [19]. Thus, tyrosinase inhibition elicited by natural compounds is the target of many research programs to counteract various disorders and cosmetic problems. Cycloorbicoside A-7-monoacetate (**2**) displayed the most potent tyrosinase inhibitory activity, estimated at 113.24 mg KAE/g, followed by the compounds **6, 7**, and **1** that showed 67.79, 64.19, and 61.24 mg KAE/g, respectively. However, compounds **12** and **14** revealed the lowest activity, estimated at 27.42 and 27.76 mg KAE/g, respectively (Figure 4).

#### 2.3.3. α-Amylase and α-Glucosidase Inhibitory Activity

Both α-amylase and α-glucosidase acted as biocatalysts to cleave glycosidic bonds (1,4) in glycogen or starch. Additionally, α-glucosidase catalyzed the last step in carbohydrate hydrolyses, resulting in the production of glucose. Regarding the inhibition of α-amylase, 3-*O*-β-d-xylopyranoside-(23*R*,24*S*)-16β,23;16α,24-diepoxycycloart-25(26)-en-3β,7β-diol (**5**) exerted the highest activity (0.55 mmol ACAE/g), followed by ursolic acid (**15**) (0.45 mmol ACAE/g) and 3-*O*-β-d-xylopyranoside-(23*R*,24*S*)-16β,23, and then 16α,24-diepoxycycloart-25(26)-en-3β,7β-diol-7–monoacetate (**4**) (0.42 mmol ACAE/g). However, 3-*O*-β-d-xylopyronoside-20*R*-25-norcycloartan-3β,7β,16β-triol-20,24-olide (**9**) revealed the lowest activity (0.13 mmol ACAE/g).

Concerning α-glucosidase, 3-*O*-β-d-xylopyranoside-(23*R*,24*S*)-16β,23;16α,24-di-epoxycycloart-25(26)-en-3β,7β-diol (**3**) showed the highest activity (25.18 mmol ACAE/g). On the contrary, cycloorbicoside A (**1**), cycloorbicoside A-7-monoacetate (**2**), 3-*O*-β-d-xylopyronoside-20*R*-25-norcycloartan-3β,7β,16β-triol-20,24-olide (**9**), cycloorbicoside B (**10**), and cyclosiversioside H (**13**) exerted no effect on the inhibition of α-glucosidase. Additionally, the remaining compounds displayed inhibitory potentials from 19.36 to 24.90 mmol ACAE/g (Table 2).

All triterpenes showed acetylcholinesterase (AChE) and butyrylcholinesterase (BChE) inhibitory potential. Additionally, some showed inhibitory activity towards tyrosinase, α-amylase, and α-glucosidase to different degrees. Enzyme inhibitory therapeutic strategies are the most promising and established strategies for alleviating disorders, including diabetes mellitus and Alzheimer′s disease, that are dramatically increasing in modern times. Patients who have Alzheimer’s disease possess low levels of acetylcholine, an important neurotransmitter of the brain. Thus, inhibition of acetylcholinesterase (AChE) that catalyzes acetylcholine hydrolysis is used to treat Alzheimer’s disease to some extent. The use of enzymes that play a role in carbohydrate digestion, such as α-amylase and α-glucosidase, is among the promising strategies for natural products and related compounds to control blood glucose levels associated with diabetes mellitus [19,20]. Many cycloartane-type triterpenes have been reported to possess antioxidant and α-glucosidase inhibitory activity, such as mangiferolic acid isolated from propolis collected by the Indonesian stingless bee (*Tetragonula sapiens* Cockerell) propolis [21]. In addition, cycloartane glycosides isolated from *Astragalus plumosus* var. *krugianus* revealed potent protective effects against oxidative stress [22]. Furthermore, one new cycloartane triterpene, namely (22*Z*,24*E*)-3β-hydroxycycloart-14,22,24-trien-26-oic acid isolated from *Garcinia hombroniana* bark, displayed potent cholinesterase inhibitory potential versus both acetylcholinesterase and butyrylcholinesterase [23]. New cycloartane-type triterpenoids isolated from the *Amberboa ramosa* entire plant revealed considerable tyrosinase inhibitory potential, together with those isolated from *Astragalus*, and thus could serve as skin-whitening agents in pharmaceuticals or cosmeceuticals [24,25].

### 2.4. Molecular Modelling Study

**Figure 5:**
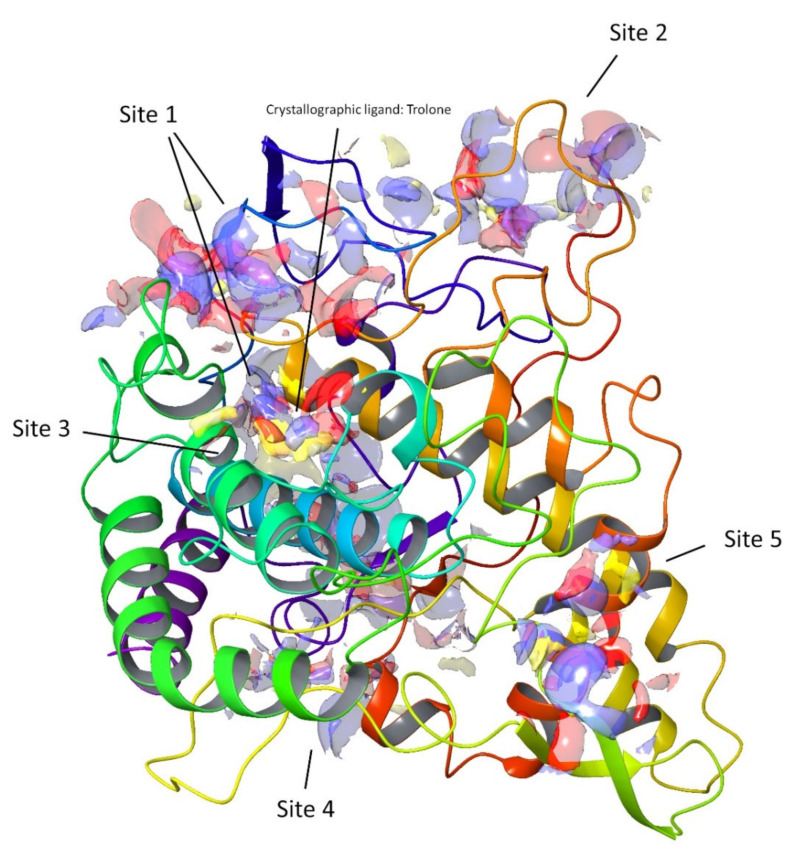
Top-ranked potential receptor-binding sites with hydrophobic (yellow), hydrogen-bond donor (blue), and hydrogen-bond acceptor (red) maps presented.

Computational techniques have been successfully used for the prediction of the ligand–target interaction [26,27]. For this study, we selected the enzyme tyrosinase as the molecular target, as some of the compounds tested in the in vitro assays demonstrated a high activity for the inhibition of this enzyme (Figure 5), e.g., compounds **2**, **6**, and **7**. In particular, compound **2** showed the highest inhibition activity among the fifteen tested compounds.

**Figure 6:**
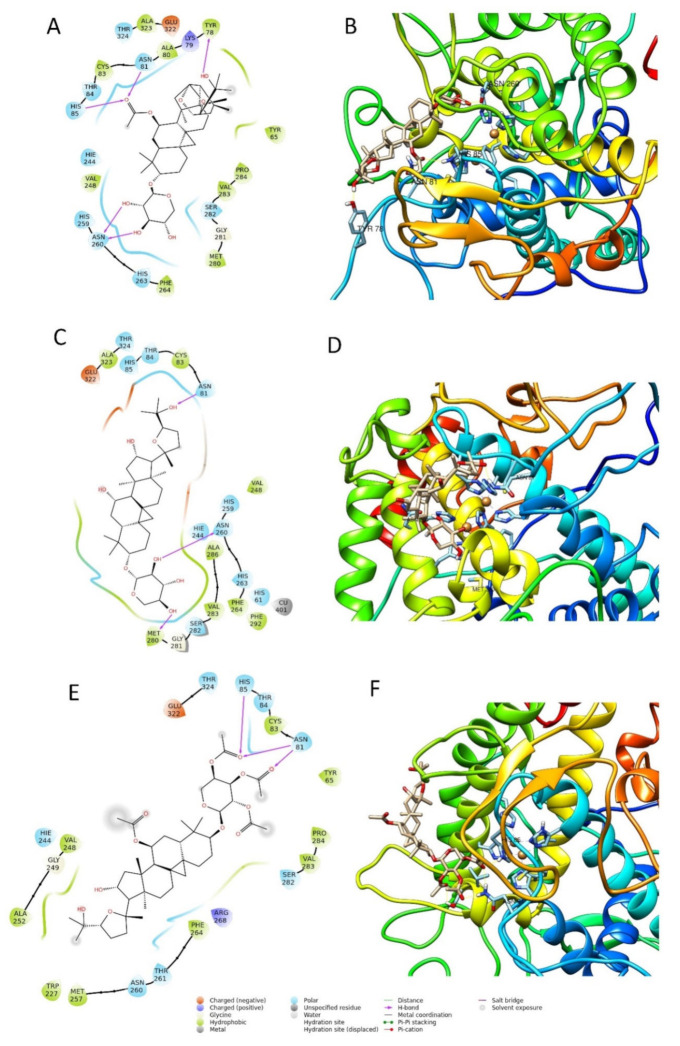
Two-dimensional and three-dimensional docking poses of compound **2** (**A**,**B**), compound **6** (**C**,**D**), and compound **7** (**E**,**F**).

In the early stage of docking performed with the standard precision method, we observed that these triterpenes according to their molecular weight, which is sensibly higher than the natural substrates of tyrosinase, such as l-dopa and tyrosine, cannot penetrate in the depth of the enzymatic pocket. Thus, in order to better understand which could be the most plausible interaction site for this class of molecules, the enzyme was subjected to a binding site search by the Sitemap routine included in Maestro [28]. This experiment returned five top-ranked potential receptor binding sites, as depicted in Figure 5. For each of the sites a docking grid was constructed and the three selected compounds **2**, **6**, and **7** were docked onto them. The best docking values were obtained for site1, which is the site containing the enzymatic cavity and surrounding area on the surface of the enzyme. Thus, site1 (Figure 6) was taken as a reference binding site and all the substances were docked with the extra precision method. After the docking calculation, the compounds revealed a docking score ranking in agreement with the in vitro data, in which compound **2** was more active than the other two. Additionally, the calculation of the DG free-binding energy was calculated by the MM-GBSA method included in Maestro and the results are also in agreement with the in vitro assay (Figure 4).

The best pose of compound **6**, with a docking score of −6.771, was able to form three interactions with binding site1 of the enzyme, namely one hydrogen bond to Asn260, one to Asn81, and one to Met280. The ΔG free energy was the lowest among the three tested compounds, equal to −22.238 Kcal/mol. The best pose of compound **7** had the lowest docking score of the series with a value of −4.689. The ΔG free-binding energy was intermediate with a value of −34.286 Kcal/mol. This molecule was found to form with the enzyme three hydrogen bonds, two to Asn81, and one to His85. Compound **2** was the most active compound, which also obtained the best docking score (−7.069 Kcal/mole) and could interact with the enzyme through several H-interactions with tyr78, Asn260, His85, and Asn81 as seen in Figure 6. Additionally, its MM-GBSA showed the highest score among the selected compounds, in agreement with the biological activity, equal to −43.987 Kcal/mol. The docking scores and MM-GBSA calculations of each compound are displayed in Table 3.

### 2.5. ADMET/TOPKAT Prediction

**Figure 7:**
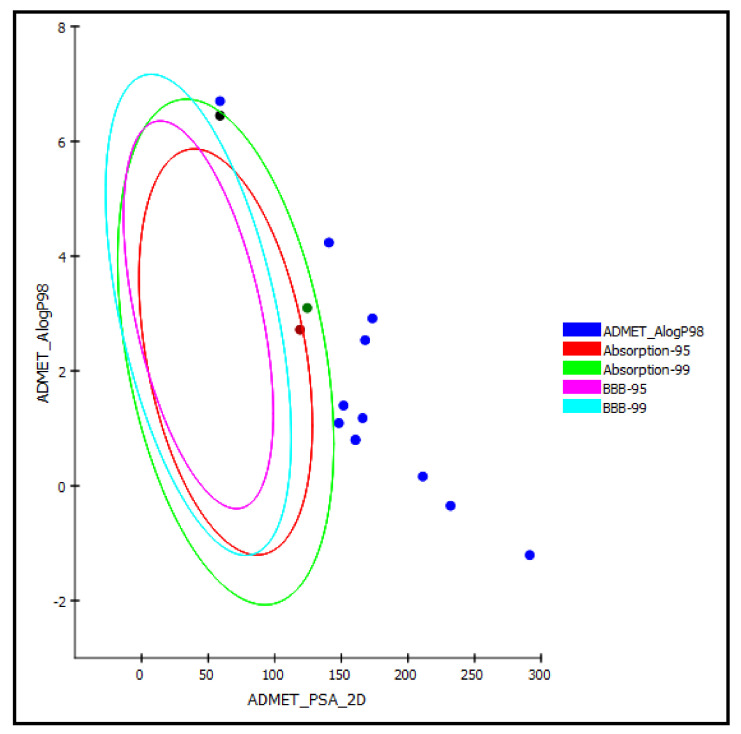
ADMET Plot of the 2D polar surface area (PSA_2D) against calculated ALogP98 for examined triterpenes, showing the 95% and 99% confidence limit ellipses corresponding to the blood–brain barrier (BBB) and to the human intestinal absorption models; compound **4** (green dot), compound **5** (red dot), and compound **14** (black dot) are (just) within acceptable boundaries for some penetration in ADMET_AlogP98.

Regarding the ADMET prediction, the pharmacokinetic and pharmacodynamic properties of the tested triterpenes were evaluated. Compound **5** was estimated to provide a high human intestinal absorption level, whereas compounds **4** and **14** in this model showed a moderate human intestinal absorption level and thus remained within the 99% absorption ellipse, as shown in the ADMET plot (Figure 7). Meanwhile, the rest of the compounds revealed low human intestinal absorption levels and likely would require certain semisynthetic modifications or special formulations to increase their absorption to achieve sufficient activity. Regarding solubility, compounds **1** and **6–10** displayed good solubility levels in contrast to the rest of the compounds that exerted low solubility. However, the prediction of the penetration of all the compounds through the BBB (blood–brain barrier) was undefined, taking level 4, and thus, as illustrated in the ADMET plot in Figure 7, all of the compounds were outside the 99% BBB confidence ellipse.

Additionally, the free drug concentration is a critical factor in the evaluation of pharmaceutical activity; thus, the probable binding of compounds to plasma protein should be determined. All triterpenes are expected to have less than 90% PPB. Cytochrome P450 2D6 (CYP2D6) is an important enzyme in the metabolism of many xenobiotics and thus its inhibition may trigger uncontrolled drug–drug interactions or drug lifetimes. Hence, evaluation of the CYP2D6 inhibition is a crucial part of the process of drug discovery and development. All of the examined triterpenes were considered non-inhibitors of CYP2D6. Additionally, they showed no hepatotoxicity in the Discovery Studio 4.5 hepatotoxic model that postulates the occurrence of dose-dependent human hepatotoxicity (Table 4).

Regarding the TOPKAT prediction, all of the tested compounds revealed to be non-mutagenic concerning the prediction of chemical Ames mutagenicity performed in silico. Additionally, all showed no carcinogenicity in both male and female rat NTP (National Toxicology Program) models, performed by Discovery Studio 4.5 in silico, except oleanolic acid (**14**) which revealed potential carcinogenicity to male rat NTP. They displayed rat oral LD50 ranging between 0.98 and 7.88 g/kg of body weight, wherein the lowest LD50 was exerted by compound **3** (0.98 g/kg of body weight) and the highest LD50 (7.88 g/kg of body weight) was displayed by **13.** Regarding skin irritancy, all of the tested compounds revealed mild skin irritancy, except compounds **6, 9,** and **13–15** that displayed moderate irritation to the skin. Regarding ocular irritation, compounds **2–5** and **9** showed no ocular irritation. In contrast, severe irritation was predicted for the aglycones oleanolic acid (**14**) and ursolic acid (**15**). However, all of the other compounds showed only moderate irritation. Thus, from the ADMET/TOPKAT prediction, compounds **4** and **5** are expected to display the best pharmacokinetic and pharmacodynamic behavior with no mutagenic, carcinogenic, or irritant effects, combined with low LD50. They also revealed considerable activity in most of the examined activities. In other cases, triterpenes and triterpene glycosides may require modifications to enhance their pharmacokinetics and pharmacodynamics, particularly those that revealed high activity (Table 5).

## 3. Materials and Methods

### 3.1. Triterpenes Used in This Study

Triterpenes and their derivatives (**1–11** and **13**) obtained from the Institute of the Chemistry of Plant Substances (Tashkent, Uzbekistan) were used in this study with purities > 95%. Compounds **12** and **14–15** were purchased from Merck, Darmstadt, Germany. They were cycloorbicoside A (**1**), cycloorbicoside A-7-monoacetate (**2**), 3-*O*-β-d-xylopyranoside-(23*R*,24*S*)-16β,23;16α,24-di-epoxycycloart-25(26)-en-3β,7β-diol 2′,3′,4′,7-tetraacetate (**3**), 3-*O*-β-d-xylopyranoside-(23*R*,24*S*)-16β,23; 16α,24-diepoxycycloart-25(26)-en-3β,7β-diol 7–monoacetate (**4**), 3-*O*-β-d-xylopyranoside-(23*R*,24*S*)-16β,23;16α,24-diepoxycycloart-25(26)-en-3β,7β-diol (**5**), cycloalpioside D (**6**), cycloalpioside D-2′,3′,4′,7-tetraacetate (**7**), cycloalpioside D -2′,3′,4′-triacetate (**8**), 3-*O*-β-d-xylopyronoside-20*R*-25-norcycloartan-3β,7β,16β-triol-20,24-olide (**9**), cycloorbicoside B (**10**), cyclosiversioside E (**11**), astragaloside IV (syn. cyclosieversioside F, astrasieversianin XIV) (**12**), cyclosiversioside H (**13**), oleanolic acid (**14**), and ursolic acid (**15**).

### 3.2. In Vitro Assays for the Evaluation of the Antioxidant Activity of the Studied Triterpenes

#### 3.2.1. Total Antioxidant Activity by the Phosphomolybdenum Method

The total antioxidant activity for all the studied compounds was determined by using the phosphomolybdenum assay and adopting a protocol described by Zengin et al. (2015) [28]. In total, 0.3 mL of each compound studied was added to 3 mL of the reagent solution containing 4 mM of ammonium molybdate, 28 mM of sodium phosphate, and 0.6 M of sulfuric acid. After incubation for 90 min at 95 °C, the absorbance of the sample was measured at 695 nm. The Trolox equivalent (mg TE/g) was used to express the total antioxidant capacity.

#### 3.2.2. 1,1-Diphenyl-2-Picrylhydrazyl (DPPH*) Radical Scavenging Capacity Assay

The DPPH* radical scavenging capacity of the examined samples was determined by adopting the method previously described by Sarikurkcu et al., (2011) [29,30]. In total, 1 mL of the tested sample was added to 4 mL of the DPPH* methanol solution (0.004%). After incubation of the samples in the dark at room temperature for 30 min, the absorbance of the sample was determined at 517 nm. The Trolox equivalent (mg TE/g) was used to express the DPPH* radical scavenging activity.

#### 3.2.3. 2,2′-Azinobis(3-Ethylbenzothiazoline)-6-Sulfonic Acid (ABTS) Cation Radical Scavenging Activity

As previously described by Re et al. (1999), ABTS cation radical scavenging activity was evaluated, accompanied by certain modifications [31]. The reaction of 2.45 mM of potassium persulfate with 7 mM of ABTS+* solution resulted in the direct production of the ABTS+* radical cation. This reaction occurs at room temperature in the dark for 12–16 h. Dilution of the ABTS+* solution using methanol was performed before starting the assay to reach an absorbance of 0.700 ± 0.02 at 734 nm. In total, 1 mL of the sample was combined and mixed with 2 mL of the prepared ABTS+* solution. After incubation of the samples at room temperature for 30 min, the absorbance of the sample was determined at 734 nm. The Trolox equivalent (mg TE/g) was used to express the ABTS radical cation scavenging activity.

#### 3.2.4. Cupric Ion-Reducing Activity (CUPRAC) Assay

The cupric ion-reducing activity (CUPRAC) was performed by following a protocol reported by Zengin et al. (2014) [32]. In total, 0.5 mL of the sample was added to a reaction mixture composed of NH_4_Ac buffer (1 mL, 1 M, pH 7.0), neocuproine (1 mL, 7.5 mM), and CuCl_2_ (1 mL, 10 mM). Similarly, the blank solution was prepared by adding 0.5 mL of the sample to a reaction mixture free from CuCl_2_. After incubation of the samples at room temperature for 30 min, the absorbance of both the sample and blank was measured at 450 nm, and then subtraction of the blank absorbance from the sample was performed. The Trolox equivalent (mg TE/g) was used to express CUPRAC activity.

#### 3.2.5. Ferric-Reducing Antioxidant Power (FRAP) Assay

The FRAP assay was performed as reported by Aktumsek et al., accompanied by certain modifications [33]. In total, 0.1 mL of the sample was added to 2 mL of the FRAP reagent, which was composed of 10 mM of 4,6-tris(2-pyridyl)-s-triazine (TPTZ) and acetate buffer (0.3 M, pH 3.6) premixed with 20 mM of ferric chloride and HCl (40 mM) in a ratio of 1:10:1 (*v/v/v*). After incubation of the samples at room temperature for 30 min, the absorbance of the sample was measured at 593 nm. The Trolox equivalent (mg TE/g) was used to express FRAP activity.

#### 3.2.6. Metal-Chelating Activity on Ferrous Ions

The metal-chelating activity on ferrous ions was measured as previously reported by Aktumsek et al. [33]. In total, 2 mL of the tested samples were mixed with 0.05 mL of 2 mM of ferric chloride solution. The initiation of the reaction was achieved via the addition of 0.2 mL of ferrozine (5 mM). Similarly, the blank preparation was done by adding 2 mL of the tested samples to 0.05 mL of 2 mM of ferric chloride solution and 0.2 mL of water without ferrozine. After incubation of the samples at room temperature for 10 min, the absorbance of both the sample and blank was measured at 562 nm, and then subtraction of the blank absorbance from the sample was performed. The EDTA equivalent (mg EDTAE/g) was used to express the metal-chelating activity.

### 3.3. In Vitro Assays for the Evaluation of the Enzyme Inhibitory Activity of the Studied Triterpenes

#### 3.3.1. Cholinesterase (ChE) Inhibitory Activity

The ChE inhibitory potential was determined using Ellman’s method as described by Aktumsek et al., accompanied by certain modifications [33]. In total, 50 μL of the tested samples were added to 125 μL of DTNB and 25 μL of acetyl (AChE) or butyryl cholinesterase (BChE) in 25 μL of Tris–HCl buffer (pH 8.0) in a 96-well microplate, followed by their incubation at 25 °C for 15 min. The reaction was initiated via the addition of 25 μL of acetylthiocholine iodide (ATCI) or butyrylthiocholine chloride (BTCl). Similarly, blank preparation was done by mixing the samples with all of the reagents previously mentioned, except for the enzyme solutions (AChE or BChE). After incubation of the samples at 25 °C for 10 min, the absorbance of both the sample and blank was measured at 405 nm, and then subtraction of the blank absorbance from the sample was performed. The Galanthamine equivalent (mg GALAE/g) equivalent was used to express the cholinesterase inhibitory activity. Note: (Absorbance (A) = 0.0607 (μg galanthamine) + 0.4746, (R2 = 0.9404) for AChE and A = 1.5806 (μg galanthamine) + 0.2839, (R2 = 0.9993) for BuChE) [19,34].

#### 3.3.2. Tyrosinase Inhibitory Activity

The tyrosinase inhibitory potential was determined by applying the modified dopachrome assay using l-DOPA as a substrate, as described by Zengin et al. (2014) [32], accompanied by certain modifications. In total, 25 μL of the sample was added to 40 μL of tyrosinase solution and 100 μL of phosphate buffer (pH 6.8) in a 96-well microplate, followed by their incubation at 25 °C for 15 min. Initiation of the reaction was done via the addition of 40 μL of l-DOPA. Similarly, preparation of blank sample was done by mixing the sample with all of the reagents previously mentioned, except for the enzyme solution. After incubation of the samples at 25 °C for 10 min, the absorbance of both the sample and blank was measured at 492 nm, and then subtraction of the blank absorbance from the sample was performed. The Kojic acid (mg KAE/g) equivalent was used to express the tyrosinase inhibitory activity, where A = 0.0775 (μg kojic acid) + 0.0163, R2 = 0.9974).

#### 3.3.3. α-Amylase Inhibitory Activity

The α-Amylase inhibitory activity was employed using the Caraway–Somogyi iodine/potassium iodide (IKI) assay as previously reported by Lazarova et al., 2015 [35], accompanied by certain modifications. In total, 25 μL of the sample was added to 50 μL of α-amylase solution in phosphate buffer (pH 6.9) with sodium chloride (6 mM) in a 96-well microplate, followed by their incubation at 37 °C for 10 min. Initiation of the reaction was done via the addition of 50 μL of starch solution. Similarly, the preparation of the blank was done by mixing the sample with all of the reagents previously mentioned, except for the enzyme solution. After incubation of the samples at 37 °C for 10 min, stopping of the reaction was achieved by the addition of 25 μL of 1 mM of HCl, that was consequently followed by the addition of 100 μL of iodine-potassium iodide solution. The absorbance of both the sample and blank was measured at 630 nm, and then subtraction of the blank absorbance from the sample was performed. The Acarbose (mg ACAEs/g) equivalent was used to express α-amylase inhibitory activity, where A = 0.9094 (mg acarbose) + 1.2921, R2 = 0.9979).

#### 3.3.4. α-Glucosidase Inhibitory Activity

Determination of the α-glucosidase inhibitory activity was achieved by employing the assay previously reported by Lazarova et al., 2015 [35], accompanied by certain modifications. In total, 50 μL of the sample was added to 50 μL of glutathione and 50 μL of enzyme solution in phosphate buffer (pH 6.8) with 50 μL of PNPG in a 96-well microplate, followed by their incubation at 37 °C for 15 min. Similarly, blank preparation was done by mixing the sample with all of the reagents previously mentioned, except for the α-glucosidase enzyme solution. Stopping of the reaction was achieved by the addition of 50 μL of 0.2 mM of HCl. The absorbance of both the sample and blank was measured at 400 nm, and then subtraction of the blank absorbance from the sample was performed. The acarbose (mg ACAE/g) equivalent was used to express α-glucosidase inhibitory activity, where A = 2.1183 (mg acarbose) – 0.2336, R2 = 0.9410.

### 3.4. Molecular Modelling Study

#### 3.4.1. Preparation of the Enzyme

The mushroom tyrosinase structure (PDB ID 2Y9X; 2.78 Å) [36] was selected for the docking experiments considering the in vitro assays which revealed very good inhibitory activity on this target, and thus was more relevant than for the other enzymatic targets (see Figure 3 and Figure 4). The raw crystallographic enzyme file was polished and prepared for docking by the PrepWizard module embedded in Maestro Schrödinger (2017), which was set to remove the non-catalytic water and all the other molecules included in the PDB file. The enzyme′s crystal structure was minimized at pH 7.4 by PROPKA following the well-established procedure used by our research group [37].

#### 3.4.2. Preparation of the Ligand

The in silico studies were performed on the most active compounds on tyrosinase, namely compounds **2**, **6**, and **7**, as mentioned above, which belong to the triterpenoid family. Before the docking experiments, the ligands as drawn manually through the Maestro 3D builder tool and starting from the base molecule of cyclosieversioside downloaded from the Zinc12 database with id:ZINC253916001—were prepared by the LigPrep tool placed in Maestro 10.1, neutralized at pH 7.4 by Epik, and minimized by force field OPLS3. The chemical structures of the ligand are depicted in Figure 1.

#### 3.4.3. Molecular Docking Experiments

The tyrosinase model was prepared using Maestro 10.2, protonated at neutral pH, and all the crystals errors were corrected automatically. The docking experiments were carried out by using Glide [38], firstly using the scoring function Standard Precision (SP) and then eXtra Precision (XP); the ligand was set to be flexible, whereas the enzyme structure was kept rigid. The docking grid was automatically calculated by Glide, centered on the crystallographic ligand or on the site map results (Figure 5), and was extended in a box of 26 × 26 × 26 angstrom. Both Cu atoms were indicated as suitable for coordinative bonds. The docking was set to allow for passing through the initial Glide screens’ 10,000 poses and then the software retained the first 500, on which a post-docking energy minimization was performed, after which only the best-ranked one was shown.

#### 3.4.4. Molecular Mechanics Energies Combined with Generalized Born and Surface Area Continuum Solvation (MM-GBSA)

The obtained docking poses were submitted to the binding energy estimation by the MM-GBSA method of the Prime module set in Maestro 10.1.

#### 3.4.5. Site Maps

The binding site analysis was performed using the SiteMap tool of the Schrodinger software. SiteMap identified five sites based on the site score, which includes size, volume, amino acid exposure, enclosure, contact, hydrophobicity, hydrophilicity, and donor/acceptor ratio. The search was set to recognize each possible binding site with a minimum of 15 site points; then, the grid was set to “fine” and the definition of hydrophobicity was set as “more restrictive” [39].

### 3.5. ADMET/TOPKAT Prediction

In this study, the triterpenes were subjected to ADMET prediction (absorption, distribution, metabolism, excretion, and toxicity) and toxicity prediction (TOPKAT) using Discovery Studio 4.5 (Accelrys Inc., San Diego, CA, USA). Blood–brain barrier penetration (BBB), aqueous solubility, plasma protein binding prediction (PPB), cytochrome P450 2D6, hepatotoxicity level, and human intestinal absorption (HIA) were chosen as descriptors of the ADMET prediction. Meanwhile, Ames mutagenicity, eye and skin irritation, carcinogenic effect on male and female rat NPT, and rat oral LD50 were chosen as parameters in the TOPKAT prediction to select entities with considerable pharmacokinetics behavior with concomitant low toxicity [37].

### 3.6. Statistical Analysis

All experiments were performed in triplicates and the results are expressed as mean ± SD (standard deviation). Variations among the different tested compounds were analyzed by using one-way variance analysis (ANOVA), followed by Tukey′s honest significant difference post-hoc test with = 0.05 using the SPSS version 14.0 program. Graphs were drawn using GraphPad Prism 5 (GraphPad Software Inc., San Diego, CA, USA).

## 4. Conclusions

Triterpenes represent a large category of naturally occurring compounds possessing many significant biological activities sometimes in the form of aglycones but often only or more specifically in the form of various glycosides. There is an increasing interest in natural triterpenoids due to their outstanding biological activities, as exemplified by their anticancer, antiviral, bactericidal, spermicidal, anti-allergic, fungicidal, and cardiovascular protective effects. The antioxidant and enzyme inhibitory potential of fifteen triterpenes of the cycloartane type was investigated using different assays. Most of the tested compounds showed antioxidant activities, although they did not contain the typical phenolic or catecholic moieties found, e.g., in plant flavonoids. The different assays reflected also different modes of antioxidant activity. All triterpenes showed AChE and BChE inhibitory potential, likely due to a general binding in the large lipophilic pocket and because of variable inhibitory activities towards α-amylase, α-glucosidase, and tyrosinase enzymes, with the latter further supported by in silico studies. While most glycoside effects, similar to those on the cholinesterases, appeared to be unspecific, tyrosinase inhibition showed clear differentiation between different compounds and appeared to be promising, especially since these lipo and amphiphilic triterpenes could show sufficient skin penetration for activity. From the ADMET/TOPKAT prediction, it can be concluded that 3-*O*-β-d-xylopyranoside-(23*R*,24*S*)-16β,23;16α,24-diepoxycycloart-25(26)-en-3β,7β-diol (**5**) and its 7-acetate (**4**) are likely to display the best pharmacokinetic and pharmacodynamic behavior with no mutagenic, carcinogenic, or irritant effects to be expected, as well as with low LD50. However, the most active substances in the in vitro test revealed to be compound **2**, **6**, and **7** for tyrosinase, while compounds **3–8** were moderate inhibitors of α-glucosidase, with scarce inhibition on α-amylase, and compounds **1–7** and **9** were moderately active on AChE. Overall, these triterpenes reveal some general inhibitory activity but also some have more specific effects (somewhat similar to another large natural products group, specifically the flavonoids, which also have some general, e.g., antioxidant, but also sometimes very specific, effects). Semisynthetic modification of such triterpenes can help to enhance both their specificity and their pharmacokinetic and pharmacodynamic properties. In particular, those that revealed high basic activity can be employed as leads for the mild natural product-based (co-)treatment of early stage Alzheimer′s disease and diabetes, and is the most promising against skin hyperpigmentation. It should be noted here that the inhibitors of tyrosinase are often used for skin treatment and are administered by topical applications, thus poor pharmacokinetic properties of compound **2** should be not considered as a limit regarding its development as an antityrosinae agent.

## Figures and Tables

**Table 1:** Antioxidant activity of fifteen triterpenes, employing the phosphomolybdenum method, and metal-chelating activity on ferrous ion assays.

Compounds	Phosphomolybenum (mmol TE/g)	Metal-Chelating (mg EDTAE/g)
Cycloorbicoside A (**1**)	1.82 ± 0.04	18.54 ± 0.06
Cycloorbicoside A-7-monoacetate (**2**)	2.26 ± 0.22	41.00 ± 0.37
3-*O-*β*-*d-Xylopyranoside-(23*R*,24*S*)-16β,23;16α,24-diepoxycycloart-25(26)-en-3β,7β-diol 2′,3′,4′,7-tetraacetate (**3**)	1.98 ± 0.06	18.16 ± 0.72
3-*O-*β-d-Xylopyranoside-(23*R*,24*S*)-16β,23;16α,24-diepoxycycloart-25(26)-en-3β,7β-diol-7-monoacetate (**4**)	1.34 ± 0.01	21.88 ± 0.96
3-*O-*β-d-Xylopyranoside-(23*R*,24*S*)-16β,23;16α,24-diepoxycycloart-25(26)-en-3β,7β-diol (**5**)	0.80 ± 0.01	15.64 ± 0.28
Cycloalpioside D (**6**)	4.05 ± 0.01	19.03 ± 0.07
Cycloalpioside D-2′,3′,4′,7-tetraacetate (**7**)	1.58 ± 0.16	25.38 ± 0.08
Cycloalpioside D-2′,3′,4′-triacetate (**8**)	1.78 ± 0.02	19.26 ± 0.33
3-*O-*β-d-Xylopyranoside-20*R*-25-norcycloartan-3β,7β,16β-triol-20,24-olide (**9**)	0.45 ± 0.02	20.19 ± 0.21
Cycloorbicoside B (**10**)	1.49 ± 0.17	4.68 ± 0.08
Cyclosieversioside E (**11**)	1.90 ± 0.16	8.33 ± 0.24
Astragaloside IV (**12**)	1.30 ± 0.04	8.73 ± 0.04
Cyclosieversioside H (**13**)	0.83 ± 0.06	3.96 ± 0.14
Oleanolic acid (**14**)	0.20 ± 0.01	3.05 ± 0.14
Ursolic acid (**15**)	0.42 ± 0.10	30.17 ± 0.07

Values are reported as mean ± S.D.; TE: Trolox equivalents; and EDTAE: EDTA equivalents.

**Table 2:** α-Amylase and α-glucosidase inhibitory activity of fifteen triterpenes expressed in mmol ACAE/g.

Compounds	α-Amylase	α-Glucosidase
Cycloorbicoside A (**1**)	0.14 ± 0.02	NA
Cycloorbicoside A-7-monoacetate (**2**)	0.33 ± 0.02	NA
3-*O-*β*-*d-Xylopyranoside-(23*R*,24*S*)-16β,23;16α,24-diepoxycycloart-25(26)-en-3β,7β-diol 2′,3′,4′,7-tetraacetate (**3**)	0.29 ± 0.01	25.18 ± 0.02
3-*O-*β-d-Xylopyranoside-(23*R*,24*S*)-16β,23;16α,24-diepoxycycloart-25(26)-en-3β,7β-diol-7-monoacetate (**4**)	0.42 ± 0.06	24.90 ± 0.09
3-*O-*β-d-Xylopyranoside-(23*R*,24*S*)-16β,23;16α,24-diepoxycycloart-25(26)-en-3β,7β-diol (**5**)	0.55 ± 0.04	24.87 ± 0.12
Cycloalpioside D (**6**)	0.35 ± 0.06	24.08 ± 1.06
Cycloalpioside D-2′,3′,4′,7-tetraacetate (**7**)	0.33 ± 0.06	24.73 ± 0.04
Cycloalpioside D-2′,3′,4′-triacetate (**8**)	0.17 ± 0.01	24.82 ± 0.05
3-*O-*β-d-Xylopyranoside-20*R*-25-norcycloartan-3β,7β,16β-triol-20,24-olide (**9**)	0.13 ± 0.09	NA
Cycloorbicoside B (**10**)	0.25 ± 0.02	NA
Cyclosieversioside E (**11**)	0.27 ± 0.01	NA
Astragaloside IV (**12**)	0.22 ± 0.03	19.36 ± 0.01
Cyclosieversioside H (**13**)	0.29 ± 0.01	NA
Oleanolic acid (**14**)	0.29 ± 0.01	20.02 ± 0.07
Ursolic acid (**15**)	0.45 ± 0.01	21.42 ± 0.01

Values are reported as mean ± S.D.; ACAE: Acarbose equivalent; and NA: not active.

**Table 3:** Docking scores and MM-GBSA values of the selected triterpenoid ligands expressed in Kcal/mol.

Compounds	Docking Scores	MM-GBSA ΔG-Binding
Cycloorbicoside A-7-monoacetate (**2**)	−7.069	−43.987
Cycloalpioside D (**6**)	−6.771	−34.286
Cycloalpioside D-2′,3′,4′,7-tetraacetate (**7**)	−4.698	−22.238

**Table 4:** The absorption, distribution, metabolism, excretion, and toxicity (ADMET) predictions for the fifteen selected triterpenes.

Compounds	Absorption Level	Solubility Level	BBB Level	PPB Level	CPY2D6	Hepatotoxic	PSA-2D	Alog p98
Cycloorbicoside A (**1**)	3	3	4	False	NI	NT	160.613	0.799
Cycloorbicoside A-7-monoacetate (**2**)	3	2	4	False	NI	NT	166.028	1.178
3-*O-*β*-*d-Xylopyranoside-(23*R*,24*S*)-16β,23;16α,24-diepoxycycloart-25(26)-en-3β,7β-diol 2′,3′,4′,7-tetraacetate (**3**)	2	2	4	False	NI	NT	140.643	4.235
3-*O-*β-d-Xylopyranoside-(23*R*,24*S*)-16β,23;16α,24-diepoxycycloart-25(26)-en-3β,7β-diol-7-monoacetate (**4**)	1	2	4	False	NI	NT	124.397	3.097
3-*O-*β-d-Xylopyranoside-(23*R*,24*S*)-16β,23;16α,24-diepoxycycloart-25(26)-en-3β,7β-diol (**5**)	0	2	4	False	NI	NT	118.982	2.718
Cycloalpioside D (**6**)	3	3	4	False	NI	NT	151.683	1.398
Cycloalpioside D-2′,3′,4′,7-tetraacetate (**7**)	3	3	4	False	NI	NT	173.344	2.915
Cycloalpioside D-2′,3′,4′-triacetate (**8**)	3	3	4	False	NI	NT	167.929	2.536
3-*O-*β-d-Xylopyranoside-20*R*-25-norcycloartan-3β,7β,16β-triol-20,24-olide (**9**)	2	3	4	False	NI	NT	148.168	1.092
Cycloorbicoside B (**10**)	3	3	4	False	NI	NT	160.63	0.162
Cyclosieversioside E (**11**)	3	2	4	False	NI	NT	211.174	−0.348
Astragaloside IV (**12**)	3	2	4	False	NI	NT	231.99	−1.207
Cyclosieversioside H (**13**)	3	2	4	False	NI	NT	291.481	6.447
Oleanolic acid (**14**)	1	1	4	False	NI	NT	58.931	6.699
Ursolic acid (**15**)	2	1	4	False	NI	NT	58.931	0.799

Note that 0, 1, 2, and 3 indicates good, moderate, low, and very low absorption, respectively; 0, 1, 2, 3, 4, and 5 indicates extremely low, very low but possible, low, good, optimal, and too high solubility, respectively; 0, 1, 2, 3, and 4 denote very high, high, medium, low, and undefined penetration through the BBB, respectively. FALSE means less than 90% and TRUE means more than 90%. in PPB (plasma protein binding) NI: non-inhibitor; NT: non-toxic; PSA 2D: 2D polar surface area; and AlogP98: the logarithm of the partition coefficient between *n*-octanol and water.

**Table 5:** TOPKAT analysis of the fifteen selected triterpenes.

Compounds	Ames Prediction	Rat Oral LD50 g/kg of Body Weight	Skin Irritancy	Ocular Irritancy	Female Rat NTP	Male Rat NTP
Cycloorbicoside A (**1**)	Non-mutagen	2.18	Mild	Moderate	Non-carcinogen	Non-carcinogen
Cycloorbicoside A-7-monoacetate (**2**)	Non-mutagen	2.06	Mild	None	Non-carcinogen	Non-carcinogen
3-*O-*β*-*d-Xylopyranoside-(23*R*,24*S*)-16β,23;16α,24-diepoxycycloart-25(26)-en-3β,7β-diol 2′,3′,4′,7-tetraacetate (**3**)	Non-mutagen	0.98	Mild	None	Non-carcinogen	Non-carcinogen
3-*O-*β-d-Xylopyranoside-(23*R*,24*S*)-16β,23;16α,24-diepoxycycloart-25(26)-en-3β,7β-diol-7-monoacetate (**4**)	Non-mutagen	1.32	Mild	None	Non-carcinogen	Non-carcinogen
3-*O-*β-d-Xylopyranoside-(23*R*,24*S*)-16β,23;16α,24-diepoxycycloart-25(26)-en-3β,7β-diol (**5**)	Non-mutagen	1.39	Mild	None	Non-carcinogen	Non-carcinogen
Cycloalpioside D (**6**)	Non-mutagen	3.06	Moderate	Moderate	Non-carcinogen	Non-carcinogen
Cycloalpioside D-2′,3′,4′,7-tetraacetate (**7**)	Non-mutagen	1.23	Mild	Moderate	Non-carcinogen	Non-carcinogen
Cycloalpioside D-2′,3′,4′-triacetate (**8**)	Non-mutagen	1.70	Mild	Moderate	Non-carcinogen	Non-carcinogen
3-*O-*β-d-Xylopyranoside-20*R*-25-norcycloartan-3β,7β,16β-triol-20,24-olide (**9**)	Non-mutagen	2.09	Moderate	None	Non-carcinogen	Non-carcinogen
Cycloorbicoside B (**10**)	Non-mutagen	2.18	Mild	Moderate	Non-carcinogen	Non-carcinogen
Cyclosieversioside E (**11**)	Non-mutagen	5.80	Mild	Moderate	Non-carcinogen	Non-carcinogen
Astragaloside IV (**12**)	Non-mutagen	7.56	Mild	Moderate	Non-carcinogen	Non-carcinogen
Cyclosieversioside H (**13**)	Non-mutagen	7.88	Moderate	Moderate	Non-carcinogen	Non-carcinogen
Oleanolic acid (**14**)	Non-mutagen	1.12	Moderate	Severe	Non-carcinogen	Carcinogen
Ursolic acid (**15**)	Non-mutagen	0.80	Moderate	Severe	Non-carcinogen	Non-Carcinogen

## Data Availability

All data are available in this study.

## Sample Availability

Samples of some compounds are available from the authors.
